# ECMO in Refractory Septic Shock: Patient Selection, Timing and Hemodynamic Targets

**DOI:** 10.3390/jcm14227904

**Published:** 2025-11-07

**Authors:** Debora Emanuela Torre, Carmelo Pirri

**Affiliations:** 1 Department of Cardiac Anesthesia and Intensive Care Unit, Cardiac Surgery, Ospedale dell’Angelo, Mestre, 30174 Venice, Italy; 2 Department of Neurosciences, Institute of Human Anatomy, University of Padova, 35121 Padova, Italy; carmelo.pirri@unipd.it

**Keywords:** septic shock, extracorporeal membrane oxygenation, veno-arterial ECMO, multiorgan dysfunction, refractory cardiovascular failure

## Abstract

**Background:** Septic shock remains a major cause of mortality in critical care, driven by profound vasoplegia, myocardial depression and refractory circulatory collapse. Conventional therapy occasionally fails to restore adequate perfusion, leading to life-threatening multi-organ failure. **Methods**: This narrative review examines current evidence on veno-arterial extracorporeal membrane oxygenation (V-A ECMO) as a salvage strategy for refractory septic shock, focusing on the pathophysiological rationale, patient selection, timing of initiation and hemodynamic management. **Results**: Data from observational studies and registry analyses suggest that V-A ECMO may improve survival in patients with septic cardiomyopathy (SCM), with reported survival rates approaching 40% in selected adult cohorts and over 50% in pediatric populations. Early initiation, phenotype-guided selection and precise hemodynamic titration are critical to optimize outcomes. **Conclusions**: The role of ECMO in septic shock remains controversial and should be restricted to experienced centers and well-defined phenotypes. Future research must refine selection criteria, standardize support strategies and evaluate long-term functional recovery beyond survival.

## 1. Introduction

Septic shock remains one of the leading causes of death in intensive care units worldwide, with mortality rates ranging between 30% and 50% despite adherence to evidence-based management strategies [1]. According to the most recent Surviving Sepsis Campaign guidelines, the cornerstone of therapy relies on timely administration of broad-spectrum antimicrobials, adequate source control hemodynamic optimization through fluids and vasopressors and organ support [2,3]. However, in a subset of patients, these measures fail to restore adequate tissue perfusion, leading to the development of refractory septic shock, a clinical scenario characterized by escalating vasopressor requirements, progressive multi-organ dysfunction and an ominous prognosis. In this context, extracorporeal membrane oxygenation (ECMO) has become more commonly considered as a salvage option [4]. Traditionally employed in cardiogenic shock and severe respiratory failure, ECMO provides temporary mechanical support by ensuring systemic oxygen delivery and circulatory stabilization, thereby creating a window of opportunity for infection control and organ recovery [4,5,6]. The rationale for ECMO use in sepsis is grounded in the recognition that septic shock encompasses not only distributive failure but also profound myocardial depression, impaired oxygen utilization and microcirculatory collapse. These features suggest that, in selected patients, mechanical circulatory support may mitigate the mismatch between oxygen delivery and demand while conventional therapies remain insufficient [7]. Nevertheless, the application of ECMO in refractory septic shock is far from being standardized. The heterogeneity of sepsis pathophysiology, coupled with the ethical and logistical complexities of initiating an invasive and resource intensive therapy in critically ill patients with uncertain prognosis, has fueled debate within the scientific community. Observational studies and registry-based analyses have reported survival in highly selected cohorts, particularly in pediatric patients and in adults with septic cardiomyopathy, but robust evidence from randomized controlled trials is lacking [8,9,10]. Moreover, concerns persist regarding patient selection, optimal timing of initiation and long-term outcomes, underscoring the need for careful appraisal of risk and benefits on a case-by-case basis. In light of these uncertainties, the present narrative review aims to critically appraise the current evidence on the use of ECMO in refractory septic shock, with particular focus on patient selection, optimal timing of cannulation, hemodynamic targets and monitoring and clinical outcomes in both adult and pediatric populations.

## 2. Materials and Methods

A comprehensive literature search was conducted to identify relevant studies addressing the use of V-A ECMO in refractory septic shock. The search was performed in the PubMed and Scopus databases, covering works published from January 2000 to September 2025. The following keywords and medical subject headings (MeSHs) terms were used in various combinations: “extracorporeal membrane oxygenation”, “septic shock”, “refractory sepsis”. Reference lists from the selected papers and recent reviews were also screened to identify additional relevant studies. The search was limited to English-language articles and included observational cohort studies, multicenter registries, case series and clinically relevant case reports, as well as systematic and narrative reviews and meta-analyses involving either adult or pediatric patients treated with V-A ECMO for refractory septic shock. Studies primarily focused on veno-venous ECMO, post-cardiotomy or post-cardiac-arrest ECMO were excluded. When overlapping cohorts were identified, the most recent or methodologically robust publication was selected. Data extraction was performed independently by the authors, focusing on study design, patient characteristics, ECMO configuration, timing of initiation, hemodynamic management, and reported outcomes. Quantitative data on survival and complications were synthesized narratively. Discrepancies were resolved though discussion among the authors and consensus was reached for all included studies. This methodological approach ensured a comprehensive and coherent synthesis of current evidence to support clinical interpretation and decision-making.

## 3. Relevant Sections

### 3.1. Physiopathological Background

Septic shock is a complex syndrome characterized not only by vasoplegia and relative hypovolemia, but also by profound derangements in myocardial function, cellular oxygen handling, and microcirculatory homeostasis. In refractory cases, conventional support may fail to rectify these derangements, thus motivating exploration of mechanical circulatory support such as veno-arterial ECMO (V-A ECMO) [11].

#### 3.1.1. Vasoplegia and Relative Hypovolemia

Profound vasoplegia is a cardinal feature of septic shock, driven by inflammatory mediators such as nitric oxide, prostanoids and cytokines that impair vascular smooth muscle reactivity, downregulate adrenergic receptors and activate ATP-sensitive potassium channels, resulting in a marked fall in systemic vascular resistance. This vasodilatory milieu limits vasopressor efficacy and contributes to refractory shock. Concurrent depletion of endogenous vasopressin and receptor desensitization further amplify catecholamine resistance, supporting the adjunctive use of vasopressin analogues in selected cases [12,13,14].

Relative hypovolemia arises from glycocalyx degradation, endothelial barrier injury, and vascular compartment expansion due to systemic vasodilation. These processes promote capillary leak and interstitial edema, reducing effective circulating volume and venous return. Although aggressive fluid resuscitation may transiently restore hemodynamics, ongoing permeability and vasoplegia often render these measures insufficient [15,16,17].

#### 3.1.2. Myocardial Depression in Sepsis

Septic cardiomyopathy (SCM) is a reversible myocardial dysfunction occurring during sepsis, in the absence of primary coronary disease [18].

**Figure 1:**
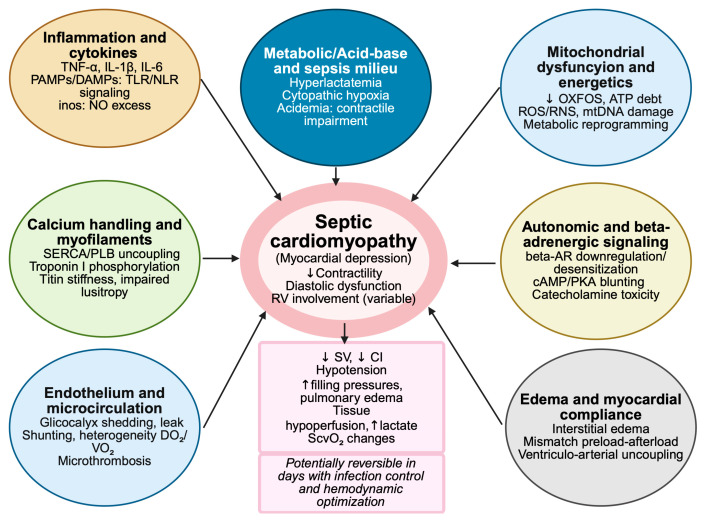
Pathophysiology of septic cardiomyopathy. OXFOS: oxidative phosphorylation; SERCA: sarcoplasmic reticulum Ca^2+^ ATPase; PLB: phospholamban; PKA: protein kinase A; SV: stroke volume; CI: cardiac index; ROS: reactive oxygen species; RNS: reactive nitrogen species; RV: right ventricle; TLR: toll-like receptor; NLR: NOD-like receptor; DO_2_: oxygen delivery; VO_2_: oxygen consumption; beta-AR: beta adrenergic receptor; NO: nitric oxide; ↑ increase, ↓ decrease. The authors generated the figure using BioRender (https://www.biorender.com/).

SCM is a frequent yet heterogenous manifestation of septic shock, which is variably reported in 10–70% of patients depending on definitions and timing [19,20]. It results from a combination of direct cytokine-mediated myocardial suppression (e.g., TNF-alpha, IL-1beta, PAMPs, DAMPs, nitric oxide), beta-adrenergic receptor downregulation, mitochondrial energetic failure, and coronary microvascular maldistribution, leading to localized ischemia and ventricular “hibernation” [21,22,23,24,25,26]. Ventriculo-arterial uncoupling further reduces cardiovascular efficiency and amplifies circulatory failure [27] (Figure 1). Importantly, SCM is often reversible once infection control and hemodynamic stability are achieved, with recovery of function typically occurring within one to two weeks [28].

#### 3.1.3. Impaired Oxygen Utilization and Cellular Dysfunction

Beyond microcirculatory and myocardial deficits, sepsis disrupts the balance between oxygen delivery (DO_2_) and utilization (VO_2_) at the cellular level. Inflammatory mediators and oxidative stress impair mitochondrial electron transport, uncouple oxidative phosphorylation, and trigger permeability transition, leading to reduced ATP generation and “cytopatic hypoxia” [29]. Even when systemic flow is restored, microvascular heterogeneity, capillary shunting, and endothelial barrier injury cause regional hypoxia and edema, increasing diffusion distance for oxygen and further limiting its cellular use. Consequently, septic shock reflects not only a deficit in oxygen delivery but also an intrinsic inability of tissue to extract and utilize oxygen effectively [30,31].

#### 3.1.4. Microcirculatory Collapse and Endothelial Injury

A central hallmark of sepsis is the disruption of microcirculatory homeostasis, affecting perfusion at the capillary and arteriole level. Endothelial activation and glycocalyx degradation increase permeability, promote leukocyte adhesion, and favor micro-thrombosis, leading to heterogeneous capillary perfusion and impaired oxygen diffusion [32,33]. Loss of vasomotor autoregulation further contributes to flow maldistribution, while platelet and coagulation activation aggravate microvascular plugins [34,35]. As a result, even when central perfusion is restored, microcirculatory derangements persist, producing regional hypoxia and impaired oxygen extraction at the tissue level.

#### 3.1.5. Mismatch Between Delivery and Demand and Phenotypes

In septic shock, the interplay of myocardial depression, microcirculatory collapse and mitochondrial dysfunction result in a profound mismatch between oxygen delivery and utilization. Reduced effective DO_2_, impaired cardiac output, and cellular hypoxia from flow heterogeneity and mitochondrial injury collectively drive progressive organ failure [36,37,38]. Increasing evidence supports the theory that septic shock is not a uniform entity but comprises distinct hemodynamic phenotypes: a distributive form dominated by vasoplegia and a cardiogenic form (SCM) characterized by severe septic cardiomyopathy. This heterogeneity is crucial for therapeutic decisions, as mechanical circulatory support such as V-A ECMO is likely to benefit only those with profound myocardial dysfunction, while patients with isolated vasoplegia derive limited hemodynamic advantage [39,40]. Messina and Vieillard-Baron proposed an echocardiographic classification distinguishing three cardiovascular phenotypes in septic shock: the “good” with preserved ventriculo-arterial coupling and adaptive hyperkinesia; the “bad”, dominated by distributive vasoplegia with preserved systolic function; the “ugly” characterized by septic cardiomyopathy and elevated filling pressures. Only patients with “ugly” phenotype are likely to benefit from temporary mechanical circulatory support such as V-A ECMO, whereas the distributive “bad” form is less amenable to such intervention [40,41]

### 3.2. Rationale for V-A ECMO in Septic Shock

Given the complex pathophysiology of septic shock, the theoretical role of V-A ECMO derives from its capacity to provide immediate circulatory support and ensure systemic oxygen delivery while underlying infection and inflammation are addressed [42], (Table 1). First, in patients with SCM myocardial dysfunction, V-A ECMO can substitute for depressed cardiac output, restoring global perfusion and oxygen transport. By providing non-pulsatile flow and maintaining systemic perfusion, ECMO creates a hemodynamic bridge that allows time for myocardial recovery, a process that is often reversible if the septic insult is controlled [8,41,42]. Second, in the context of refractory vasoplegia, the benefits of V-A ECMO are less straightforward [43]. While ECMO cannot correct the profound loss of vascular tone, it can at least guarantee a minimal level of systemic flow and perfusion pressure when vasopressor therapy is insufficient. This may prevent immediate cardiovascular collapse, although outcomes in this phenotype remain poor. Third, with regard to microcirculatory and cellular derangements, ECMO contributes by increasing global oxygen delivery (DO_2_), which may improve the gradient for oxygen diffusion across partially functional microvascular networks. Although ECMO does not directly reverse endothelial dysfunction or mitochondrial impairment, maintaining adequate systemic oxygen transport may mitigate the progression of organ injury and buy time for resolution of sepsis- induced metabolic dysfunction [44].

Finally, ECMO may serve as a platform for integrated extracorporeal therapies, such as continuous renal replacement therapy or hemoadsorption, which can be combined within the same circuit to optimize multi-organ support and inflammatory mediator removal [45,46]. Overall, the pathophysiological rationale suggests that V-A ECMO is most likely to benefit patients with a predominant cardiogenic component of septic shock, while its role in pure distributive failure remains controversial [8,42].

### 3.3. Practical Consideration: Patient Selection, Timing, and Cannulation Strategy

#### 3.3.1. Patient Selection

Identifying the subset of patients with septic shock who may derive benefit from V-A ECMO remains one of the greatest challenges. Outcomes are most favorable in patients with a predominant cardiogenic component, typically manifesting as SCM with left ventricular ejection fraction <20–25%. Conversely, patients with isolated vasoplegic phenotypes, characterized by preserved ventricular function but profound systemic vasodilation, experience poor outcomes. Additional considerations include age and comorbidity profile, evidence ongoing end-organ hypoperfusion with poor lactate clearance and treatment in high-volume ECMO centers, where outcomes are superior due to greater expertise and structured protocols [4,8,41,42,47].

#### 3.3.2. Timing of ECMO Initiation

Prolonged reliance on escalating vasopressors with ongoing organ dysfunction before cannulation is associated with poor outcomes. Across pediatric and adult reports, earlier initiation after recognition of refractory shock, before secondary organ injury becomes established, appears to yield better results, whereas delayed initiation after sustained uncorrected shock consistently fares poorly. Accordingly, ECMO should be considered expeditiously once refractory shock is confirmed and optimized conventional measures, appropriate antimicrobials and source control, fluids, vasopressors and inotropes, have clearly failed [4,8,41,48,49,50,51,52], (Box 1).

Box 1 **Definition of refractory septic shock and suggested ECMO candidacy. MAP: mean arterial pressure; ScvO_2_: central venous oxygen saturation; PvaCO_2_: venous to arterial carbon dioxide difference; LVEF: left ventricular ejection fraction.**

**Refractory septic shock**

Defined as persistent circulatory and metabolic failure despite optimized conventional therapy (adequate fluids, vasopressors, inotropes, antimicrobials and source control) with the following attributes:

- MAP < 65 mmHg despite norepinephrine ≥0.5–1 μg/kg/min ±vasopressin;
- Serum lactate >4 mmol/L or no clearance over 6 h;
- ScvO_2_ < 65% or PvaCO_2_ >6 mmHg;
- Cardiac index < 2.0 L/min/m^2^ or LVEF < 20–25%;
- Ongoing signs of hypoperfusion (oliguria, mottling, and rising creatinine).

**ECMO candidacy**

Consider V-A ECMO when refractory septic shock persists despite optimized therapy, in the presence of severe myocardial depression and a potentially controllable infection source and without irreversible comorbidities.

Discourage ECMO in isolated vasoplegia with preserved contractility.

**Timing consideration**

Early initiation, before cardiac arrest or a prolonged low-output state with multi-organ failure.

#### 3.3.3. Cannulation Strategy

The cannulation strategy must be tailored to the patient profile and anticipated duration of support:

- Peripheral femoro-femoral V-A ECMO is the most widely adopted approach due to its rapid deployability. However, it is associated with a substantial risk of distal limb ischemia, necessitating careful implementation of distal limb perfusion catheters to maintain antegrade flow to the cannulated leg. Some centers further mitigate ischemic complications by placing thinner arterial lines, such as the ankle, more distally [53,54,55]. Optimal drainage cannula positioning, ideally with the tip in the upper right atrium or lower superior vena cava, reduces the risk of severe differential oxygenation phenomena, especially in cases of lung failure [56].
- Axillary artery cannulation provides antegrade aortic flow, which improves cerebral and coronary oxygen delivery, thereby reducing the incidence of upper body hypoxemia or “Harlequin syndrome”. This approach may be particularly advantageous for prolonged ECMO support [57,58].
- Central cannulation, involving drainage from the right atrium and arterial return to ascending aorta via median sternotomy, allows for direct ventricular unloading and higher ECMO flow rates. While rarely applied in septic patients, it remains a cornerstone in post-cardiotomy scenarios, where maximal circulatory support is indicated [41,59,60].
- Hybrid VAV ECMO may be indicated in septic shock complicated by severe ARDS, offering simultaneous circulatory and respiratory support. These composite circuits demand meticulous management to prevent recirculation, coagulopathy risk associated with Y-piece connectors and flow-balancing devices and distal limb ischemia, especially during the weaning phase as circulatory support is withdrawn. The balancing of venous and arterial return flow fractions is critical to optimize efficacy and minimize complications [60,61,62].

Crucially, pre-cannulation imaging using ultrasound and echocardiography is imperative to assess vessel caliber and cardiac function, facilitating selection of appropriately sized cannulas that maximize drainage efficiency while minimizing negative suction pressures and hemodynamic instability [63].

#### 3.3.4. Intensity of Support

Recent evidence highlights the concept of ECMO support intensity as a relevant determinant of outcome in refractory septic shock [64]. Rather than a binary intervention, V-A ECMO delivers a variable hemodynamic load that must be adapted to the degree of circulatory failure and residual cardiac function. Excessive flow may worsen ventriculo-arterial uncoupling and impede myocardial recovery, whereas insufficient flow may fail to ensure adequate oxygen delivery [65]. Parameters such as the vasoactive inotropic score (VIS), ECMO flow-to-body surface area ratio and lactate clearance can provide a dynamic assessment of support adequacy. Patients requiring persistently high ECMO flow and vasopressor doses typically represent a cohort with extreme cardiovascular failure and poor prognosis. Conversely, progressive flow optimization and VIS reduction are associated with better myocardial recovery and higher weaning success [66,67,68]. This hemodynamic perspective complements timing and phenotype-based selection, emphasizing the need for individualized titration of ECMO intensity to balance circulatory support and myocardial protection.

### 3.4. Role of Echocardiography and Hemodynamic Monitoring in ECMO Decision-Making

Accurate hemodynamic assessment is pivotal for distinguishing between septic shock phenotypes and identifying candidates who may benefit from V-A ECMO [69]. Since the hemodynamic presentation of sepsis ranges from distributive vasoplegia to profound septic cardiomyopathy, a physiology-guided approach integrating echocardiographic and invasive monitoring data is essential for informed decision-making (Table 2). Critical care echocardiography represents the cornerstone for real-time characterization of cardiac performance in septic shock [40,69,70]. Transthoracic and transesophageal imaging allow quantification of left ventricular ejection fraction (LVEF), left ventricular outflow tract velocity time integral (LVOT VTI), right ventricular function (tricuspid annular plane systolic excursion, TAPSE, right ventricular fractional area change, RVFAC), diastolic parameters and dynamic assessment of ventricular filling pressures. A markedly reduced LVEF (<25%) or evidence of global hypokinesia with preserved end-diastolic volume supports the diagnosis of SCM. Conversely, hyperdynamic ventricles with normal filling and severe vasodilation suggest a distributive phenotype [40,71,72]. Advanced echocardiographic indices, including ventriculo-arterial coupling (Ea, arterial elastance/Es, end-systolic elastance ratio), myocardial strain imaging and serial stroke volume variation, provide further insights into cardiovascular efficiency and recovery potential. An Ea/Es >1.3 reflects ventriculo-arterial decoupling and identifies patients in whom contractile dysfunction predominates, a finding associated with poor response to vasopressors and potential benefit from mechanical circulatory support [26,73,74,75,76].

Complementary invasive tools, such as pulmonary artery catheterization, pulse contour analysis (PiCCO, LiDCO, Most Care), or transpulmonary thermodilution, enable continuous measurement of cardiac index, stroke volume, extravascular lung water and systemic vascular resistance. These parameters guide individualized optimization of preload, afterload, and contractility before ECMO is considered [77,78,79].

Metabolic indices, including central venous oxygen saturation (ScvO_2_), the venous-to arterial carbon dioxide gap (PvaCO_2_) and the delta CO_2_/O_2_ ratio, help identify persistent tissue hypoperfusion and oxygen extraction deficits despite maximal conventional therapy. Persistent lactate levels >4 mmol/L, ScvO_2_ < 65% and low cardiac index < 2 mL/min/m^2^), despite adequate filling and vasopressors, are pragmatic criteria suggestive of refractory shock and may trigger ECMO evaluation [80,81].

### 3.5. Clinical Evidence and Guidelines

The clinical evidence regarding V-A ECMO in refractory septic shock is derived almost exclusively from observational data, registry reports, and meta-analyses, with no randomized controlled trials available to date. Despite the physiological rationale supporting its use, outcomes remain heterogeneous, reflecting the complexity of sepsis pathophysiology and the variability in patient selection and timing of initiation.

In adult populations, survival rates are generally low, often ranging between 15% and 36% [8]. Several independent cohort studies and meta-analyses have suggested that patients with septic cardiomyopathy (SCM), characterized by left ventricular ejection fraction below 20–25% or a low cardiac index, appear to derive the greatest benefit, with survival approaching 40% in selected cohorts (Table 3). In this subgroup, ECMO can temporarily restore systemic flow, bridge the failing myocardium, and provide a window for infection control and organ recovery. Conversely, patients with predominantly distributive or vasoplegic phenotypes, despite preserved systolic function, show limited response to mechanical circulatory support, emphasizing the importance of phenotype-guided selection [4,8,42,82,83,84,85,86,87,88].

In contrast, pediatric experience is comparatively more encouraging (Table 4). Several multicenter reports and registry analyses have documented survival rates between 40% and 60%, even in patients presenting with refractory shock despite high vasopressors doses. Younger patients often demonstrate greater reversibility of septic cardiomyopathy and the earlier use of ECMO in specialized pediatric centers contributes to improved outcomes [9,89,90,91,92,93]. A recent meta-analysis highlighted a survival rate of 53% in children compared to 18% in adults, underscoring the differential impact of age and phenotype on ECMO outcomes [41].

It should be noted that these adult and pediatric survival data are not directly comparable. Pediatric cohorts generally reflect earlier ECMO initiation in high-volume centers and a lower comorbidity burden, often in the context of potentially reversible sepsis-induced myocardial dysfunction. In contrast, adult studies frequently include patients with delayed or post-cardiac arrest cannulation and more heterogeneous shock phenotypes. Therefore, the apparent difference in survival likely reflects variations in patient selection, timing, and disease reversibility rather than intrinsic differences in ECMO efficacy.

Current international guidelines provide only limited and cautious recommendation on the use of ECMO in septic shock, reflecting the paucity of high-quality evidence in this setting. The Surviving Sepsis campaign (SCC) adult guidelines do not offer a formal recommendation for the routine use of ECMO in refractory septic shock. ECMO is mentioned as a potential rescue strategy in specific scenarios, primarily for severe acute respiratory distress syndrome (ARDS) complicating sepsis, while its role in pure circulatory failure remains undefined [2,94]. This omission underscores both the scarcity of randomized data and the uncertainty surrounding patient selection and outcomes in adults with distributive shock phenotypes. By contrast, the 2020 SCC pediatric guidelines explicitly recognize V-A ECMO as a potential salvage therapy in refractory septic shock. The panel suggests considering ECMO in children who remain hemodynamically unstable despite optimized conventional therapy, including fluids, vasopressors, inotropes and appropriate source control [3]. Although this recommendation is based on low-quality evidence, it reflects the relatively better survival rates reported in pediatric cohorts and the reversibility of sepsis-induced myocardial dysfunction in younger patients. Importantly, the guidelines emphasize that ECMO should be implemented only in experienced centers with the infrastructure and multidisciplinary expertise required to manage both technical and clinical complexities. Overall, current guideline statements highlight a striking difference between adults and children: while pediatric sepsis management acknowledges ECMO as a valid last-resort option, adult recommendations remain non-committal. This divergence reflects not only differences in clinical outcomes but also the urgent need for prospective trials to clarify the role of ECMO in adult refractory septic shock.

### 3.6. Integrated Management: Antimicrobial Therapy, Source Control, and Adjunctive Extracorporeal Support

**Figure 2:**
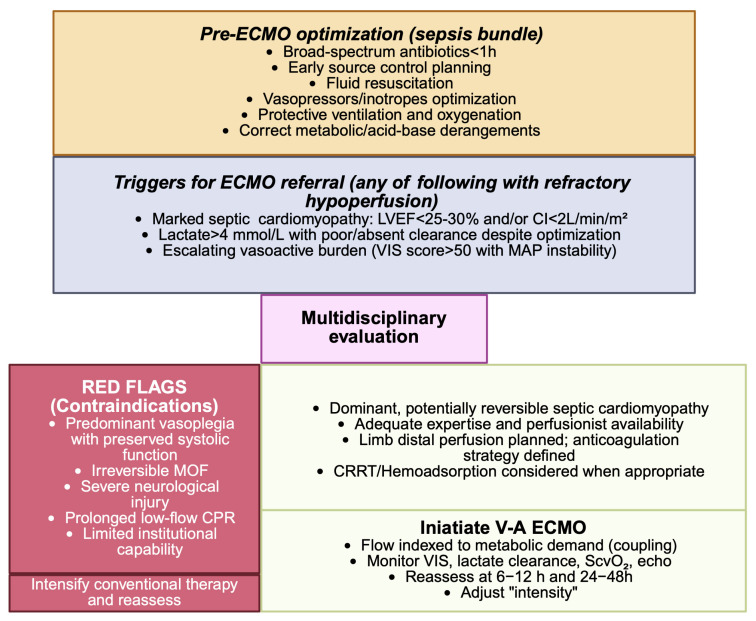
Initiation algorithm for V-A ECMO in refractory septic shock. V-A ECMO: veno-arterial extracorporeal membrane oxygenation; LVEF: left ventricular ejection fraction; CI: cardiac index; VIS: vasoactive inotropic score; MAP: mean arterial pressure; MOF: multi-organ failure; CPR: cardiopulmonary bypass; CRRT: continuous renal replacement therapy; ScvO_2_: central venous oxygen saturation. The figure was generated by the authors using BioRender.

Optimal management of refractory septic shock supported with V-A ECMO requires a multimodal strategy that extends beyond circulatory assistance (Figure 2). Antimicrobial therapy, source control, and additional extracorporeal interventions represent fundamental pillars that must be carefully integrated to maximize the chances of survival (Table 5).

Antimicrobial therapy remains the cornerstone of sepsis management. Early administration of broad-spectrum antibiotics, ideally within the first hour of shock recognition, is consistently associated with improved outcomes [95]. Under ECMO conditions, however, pharmacokinetics and pharmacodynamics of antimicrobials may be significantly altered due to sequestration within the circuit, expanded volume of distribution and organ dysfunction. These factors necessitate dose adjustments, therapeutic drug monitoring when available and close collaboration with clinical pharmacology services to ensure effective bactericidal concentration [96,97,98].

Source control is equally indispensable, as ongoing infection renders hemodynamic support futile. Surgical drainage, removal of infected devices or debridement of necrotic tissue should be pursued with urgency, even in patients on ECMO support [99]. The invasive nature of these procedures in anticoagulated patients requires meticulous perioperative planning, but timely and definitive control of the infectious focus remains a determinant of recovery.

Beyond infection management, adjunctive extracorporeal therapy plays a complementary role in stabilizing patients with multi-organ dysfunction. Continuous renal replacement therapy (CRRT) is frequently required for acute kidney injury or metabolic derangements and can be seamlessly integrated into the ECMO circuit [100]. In addition, hemoadsorption and hemoperfusion devices have been explored as potential strategies to attenuate the overwhelming inflammatory response and reduce circulating cytokine burden. While robust evidence of survival benefit is still lacking, preliminary data suggest these techniques may mitigate vasoplegia, improve hemodynamic stability, and facilitate weaning from vasopressors in selected patients [101,102]. Taken together, the integrated management of septic shock under ECMO support requires a highly coordinated, multidisciplinary approach. Circulatory support alone cannot reverse sepsis; only the synergy of timely antibiotics, effective source control, and tailored use of extracorporeal adjuncts offers a realistic chance of restoring homeostasis and organ function.

#### Pharmacokinetic Implications of ECMO on Antibiotic Therapy

The extracorporeal circuit of V-A ECMO significantly alters the pharmacokinetics of several antimicrobial agents through drug sequestration on circuit components, increased circulating volume and variable organ perfusion affecting hepatic and renal clearance. Lipophilic and highly protein-bound antibiotics (e.g., beta-lactams, fluoroquinolones, macrolides) are particularly susceptible to adsorption and distribution changes, often resulting in subtherapeutic plasma concentration during the early phase of support. Conversely, reduced hepatic metabolism or renal excretion may lead to accumulation of hydrophilic drugs once end-organ dysfunction develops. Therefore, dosing should be individualized according to pharmacodynamic targets, ECMO configuration, and renal replacement use, with a preference for extended or continuous infusion and therapeutic drug monitoring whenever available. These adjustments are critical to ensure adequate exposure and prevent treatment failure or resistance development [95,96,97,103].

### 3.7. Complications and Long-Term Outcomes

Despite technological advances, V-A ECMO remains associated with a substantial complication burden. Bleeding, thrombosis, neurological injury, limb ischemia, and nosocomial infection are the most frequent events, often amplified by the inflammatory and coagulopathic milieu of sepsis [104,105,106,107,108,109,110,111,112] (Table 6). These complications not only impact survival but also influence long-term neurological and functional recovery.

The integration of structured surveillance protocols, targeted anticoagulation, and multidisciplinary post-ICU follow-up remains essential to reduce morbidity and improve long-term recovery among survivors.

#### Long-Term Neurological and Functional Outcomes

Survivors of V-A ECMO for refractory septic shock frequently face substantial long-term sequelae extending beyond hospital discharge. Neurological injury, including hypoxic–ischemic damage, embolic stroke, and critical illness-related encephalopathy, may result in persistent cognitive and motor impairment. Moreover, post-intensive care syndrome, characterized by neuromuscular weakness, neurocognitive decline, anxiety, depression and post-traumatic stress, has been reported in up to 40–60% of ECMO survivors. Functional recovery is often slow and incomplete, with only a proportion regaining pre-morbid independence or returning to work. These data underscore that survival alone is not a sufficient endpoint; structured neurocognitive follow-up, psychological support, and tailored rehabilitation programs are essential components of post-ECMO care to restore meaningful quality of life [113,114,115].

### 3.8. Ethical and Organizational Considerations

The initiation of V-A ECMO in refractory septic shock entails complex ethical and organizational challenges extending beyond individual patient care (Table 7). Decisions must balance the moral duty to rescue with the potential for non-beneficial prolongation of treatment in case of uncertain outcomes. Transparent communication with families and surrogate decision-makers is essential to ensure informed consent and shared understanding of prognosis and possible complications. From an institutional perspective, ECMO requires specialized infrastructure, high staff-to-patient ratios and experienced multidisciplinary teams. Concentrating cases in high-volume centers consistently improves outcomes and ensures quality control. At the social level, equitable access and responsible resource stewardship remain key priorities [116,117,118].

#### Futility and Exclusion Criteria

In parallel with identifying appropriate candidates, it is equally important to recognize situations in which V-A ECMO is unlikely to provide meaningful benefit. Common “red light” features include the following:

- Devasting or irreversible neurologic injury (massive intracranial hemorrhage, severe encephalopathy);
- Irreversible or end-stage comorbidities (advanced malignancy, terminal chronic organ failure, or severe frailty);
- Established multi-organ failure with no realistic potential for recovery at the time of evaluation;
- Extreme vasoplegic shock without demonstrable myocardial depression, in which mechanical circulatory support cannot restore vascular tone;
- Prolonged unresuscitated low-flow or no-flow states preceding ECMO consideration.

In such scenarios, ECMO may only prolong the dying process without restoring physiological homeostasis. Early recognition of these exclusion criteria supports ethical proportionality, optimizes resource stewardship, and preserves the integrity of decision-making in critical care [5,119]

## 4. Discussion

The use of V-A ECMO in refractory septic shock remains a clinical and ethical challenge. Despite technological progress, clear evidence of survival benefit in adults is still lacking. Survival in adults with refractory septic shock supported by V-A ECMO ranges between 15 and 36%, reaching 40% in selected cardiogenic phenotypes, whereas pediatric outcomes are higher (45–60%), likely reflecting earlier cannulation, lower comorbidity burden and greater myocardial reversibility. The superior outcomes observed in pediatric cohorts likely result from a combination of physiological and logistical factors, including a lower comorbidity burden, greater myocardial recovery, earlier timing of cannulation and management within high-volume centers with specialized multidisciplinary expertise. These variables, rather than intrinsic differences in ECMO efficacy, largely account for observed survival advantage in children [120].

Most data derive from retrospective series and registry analyses, whose heterogeneity mirrors the biological and hemodynamic complexity of sepsis itself.

Overall, the quality of available evidence remains limited, as most studies are observational, retrospective, and subject to significant selection bias. Heterogeneity in patient population, ECMO configurations, and timing of initiation further complicates data interpretation and the lack of randomized or controlled studies precludes definitive conclusions regarding efficacy.

Consequently, the interpretation of outcomes must be nuanced, acknowledging the influence of patient phenotype, timing and center expertise. Among these determinants, the hemodynamic phenotype has emerged as the key to rational patient selection. In the context of septic cardiomyopathy, where profound but potentially reversible myocardial depression compromises systemic perfusion, ECMO may serve as a transient bridge to recovery. By contrast, patients with preserved systolic function and predominant vasoplegia derive little hemodynamic advantage, as ECMO cannot address the underlying distributive collapse. This observation underscores the central role of precision hemodynamic assessment, integrating echocardiography, invasive monitoring, and metabolic indices, in guiding indication and predicting benefit [8,40,50,51,83,85,86]. Initiating ECMO before irreversible multi-organ failure develops is essential, yet overly aggressive flow settings may impair ventriculo-arterial coupling and delay myocardial recovery. The emerging concept of ECMO intensity, defined through the interplay of flow demand, vasoactive inotropic score and lactate kinetics, offers a promising framework for titrating support to metabolic requirements. This paradigm promotes a physiology-guided strategy in which circulatory assistance is dynamically adapted rather than applied as a fixed intervention [66,67,68]. However, ECMO alone rarely alters the trajectory of refractory sepsis. Optimal outcomes require integrated management, combining prompt infection control, antimicrobial therapy and, when indicated, adjunctive extracorporeal therapies such as CRRT or hemoadsorption. Such multimodal coordination not only stabilizes hemodynamics but also facilitates the restoration of homeostasis and end-organ perfusion [2,4,33,45,46,121,122,123]. The clinical potential of ECMO must, nevertheless, be weighed against its substantial complication burden. Major complications include bleeding, thrombosis, neurologic injury, limb ischemia, and nosocomial infection, amplified by the inflammatory and coagulopathic pattern of sepsis [53,54]. Neurological sequelae and post-intensive care syndrome may persist even among survivors, limiting the number of patients who achieve full functional recovery [108,109,124]. This reality demands that ECMO deployment be accompanied by meticulous monitoring, optimized anticoagulation, and structured follow-up to assess both short- and long-term outcomes [125]. Beyond the technical domain, ethical and organizational considerations are inseparable from the discussion.

Given the invasiveness and uncertain benefit of ECMO, proportionality, potential futility and shared-decision-making with surrogates must guide initiation. Outcomes improve in high-volume centers, highlighting the importance of institutional preparedness and standardized protocols [116,117,118,126,127,128]. In synthesis, the evidence supports a selective and physiology-driven approach to ECMO in refractory septic shock. Its most plausible role lies in bridging reversible septic cardiomyopathy within an integrated care framework. While pediatric data demonstrate higher survival and clearer indications, adult evidence remains inconclusive and demands prospective, phenotype-stratified studies [9,51,86,87,88]. Future research should focus not only on whether ECMO improves patients’ condition but also on how it may foster recovery of meaningful physiological and neurocognitive function. Ultimately, the deployment of V-A ECMO for treating sepsis should reflect a balance between scientific rationale, ethical responsibility, and system sustainability. When used judiciously, within experienced centers and under precise hemodynamic guidance, it may represent not merely a rescue intervention but a carefully calibrated instrument to support life while the underlying pathology is reversed.

## 5. Conclusions and Future Directions

Current evidence confirms that the role of V-A ECMO in refractory septic shock remains uncertain and highly context-dependent. Its use should be limited to selected cases where myocardial dysfunction is potentially reversible and conventional therapy has failed. The limited survival benefit observed in adults reflects both the heterogeneity of septic shock and the lack of standardized criteria for initiation, timing, and support modulation. Future studies should adopt a phenotype-based approach, distinguishing cardiogenic from distributive septic shock, and should pursue the development of standardized initiation and management criteria to harmonize patient selection across centers. Rigorous prospective research is needed to clarify these aspects and to identify reliable physiological and biochemical markers that can guide individualized decision-making. Future efforts should also expand the focus beyond short-term survival, addressing long-term outcomes, functional recovery, and quality of life among survivors. Collaborative multicenter networks and ethically grounded frameworks for patient selection and resource allocation will be crucial to translate ECMO from an empirical rescue into a rational, evidence-based therapeutic option.

## Figures and Tables

**Table 1:** Pathophysiological mechanism in refractory septic shock and their modulation by V-A ECMO. NO: nitric oxide; CO: cardiac output; DO_2_: oxygen delivery; CRRT: continuous renal replacement therapy; MOF: multi-organ failure; ↓ decrease.

Mechanism	Pathophysiological Feature	Effect on Systemic Perfusion	Potential ECMO Contribution
Septic cardiomyopathy	Reversible myocardial depression with ↓ contractility and impaired ventriculo-arterial coupling	↓ CO ↓ DO_2_ Risk of circulatory collapse	Restores flow and oxygen transport; may allow myocardial recovery
Vasoplegia	Excess NO and inflammatory mediators cause vascular hyporesponsiveness and loss of tone	↓ Systemic vascular resistance, distributive shock	Limited benefit; ECMO does not correct vasoplegia and requires adjunctive vasopressors
Microcirculatory failure	Endothelial injury, glycocalyx degradation, capillary shunting	Regional hypoxia despite adequate macrocirculatory flow	ECMO restores oxygen delivery but may not normalize microvascular perfusion heterogeneity
Mitochondrial dysfunction	Impaired oxidative phosphorylation (“cytopatic hypoxia)	↓ cellular ATP despite normal oxygen supply; bioenergetic failure	ECMO provides oxygenation but cannot reverse intrinsic metabolic failure
Inflammatory cascade	Cytokine storm, endothelial activation, and coagulation imbalance	Microvascular thrombosis and MOF	Adjunctive extracorporeal therapies (e.g., CRRT, hemoadsorption) may attenuate inflammation

**Table 2:** Suggested hemodynamic thresholds/targets for ECMO initiation, management and weaning in refractory septic shock. LVEF: left ventricular ejection fraction; CI: cardiac index; SBP: systolic blood pressure; VIS: vasoactive–inotropic score; ScvO_2_: central venous oxygen saturation; PvaCO_2_: venous to arterial carbon dioxide difference; Ea/Es: arterial elastance/end-systolic elastance; Hb: hemoglobin.

Phase	Parameter	Threshold/Target	Clinical Implication
Initiation (consider ECMO)	LVEF	<20–25%	Indicates that a septic cardiomyopathy candidate for V-A ECMO is refractory to fluids, inotropes, vasopressors, and infection control.
	CI	<2 L/min/m^2^	Suggests inadequate forward flow despite optimized therapy.
	SBP	<90 mmHg (adult) <50 mmHg (pediatric)	Inadequate perfusion pressure and loss of vascular tone.
	Urine output	<30 mL/h (adult) <1 mL/kg/h pediatric)	End-organ hypoperfusion and renal dysfunction.
	Lactate	>4 mmol/L and not clearing within 6 h	Persistent tissue hypoperfusion.
	VIS	>40–50	Refractory shock despite maximal pharmacologic support.
	ScvO_2_	<65%	Global oxygen delivery deficit.
	PvaCO_2_ gap	>6 mmHg	Indicates poor CO_2_ washout and ongoing hypoperfusion.
Management (on ECMO)	ECMO flow (indexed)	2.2–2.6 L/min/m^2^ (adjust to perfusion markers)	Maintain adequate systemic flow while avoiding ventricular overload.
	MAP	65–75 mmHg	Support systemic perfusion; titrate vasopressors accordingly.
	Lactate trend	Decreasing >10% within 6 h or normalization within 24 h	Surrogate of effective support and metabolic recovery.
	VIS	Progressive reduction	Reflects improving native cardiac output and coupling.
	DO_2_/VO_2_	≈4–5	Indicates adequate oxygen delivery relative to metabolic demand; values <3 suggest insufficient perfusion or excessive O_2_ consumption and require optimization of flow, Hb or oxygenation.
	Ea/Es	≤1.3	Indicates restored ventriculo-arterial coupling
Weaning (readiness)	LVEF	>25–30%	Evidence of myocardial recovery.
	CI	>2.5 L/min/m^2^ (with low vasoactive support)	Adequate native output.
	Lactate	Normalized (<2 mmol/L)	Restored metabolic balance.
	ScvO_2_	≥65–70%	Adequate oxygen delivery.
	Ea/Es	≃1	Physiological ventriculo-arterial coupling restored.
	VIS	<10	Minimal pharmacologic support.

**Table 3:** Outcomes of V-A ECMO in refractory septic shock: adult population. V-A ECMO: veno-arterial extracorporeal membrane oxygenation; V-V ECMO: veno-venous extracorporeal membrane oxygenation; VAV ECMO: veno-arterial-venous extracorporeal membrane oxygenation; LVEF: left ventricular ejection fraction; CPR: cardiopulmonary resuscitation; SAPS II: simplified acute physiology score II; n.s.: not specified whether post CPR patients were included.

Study/Year	Design	n	Main Findings	Reported Survival
Ling RR et al., Crit Care 2021 [8]	Systematic review with meta-analysis (14 observational studies)	468 V-A CPR included	Best outcomes in septic cardiomyopathy; distributive shock associated with poor prognosis	36.4% (32.1% LVEF > 35%; 62% LVEF < 20%)
Choi MJ et al., Ann Thorac Surg. 2017 [47]	Retrospective observational	28 V-A (21), V-V (4), VAV (3) CPR included	A SAPS II score ≤80 is an indicator of favorable outcome	35.7%
Ro SK et al., J Thorac Cardiovasc Surg. 2018 [50]	Observational retrospective	71 V-A CPR included	Elevated arterial lactate before and after ECMO is associated with an increased risk of in-hospital mortality	15.5%
Park TK et al., Eur J cardiothorac Surg 2014 [51]	Retrospective single-center observational	32 V-A CPR included	Post-CPR status is associated with poor outcomes; ECMO is seldom beneficial	21.9%
Bréchot N et al., Crit Care Med 2013 [82]	Retrospective single-center observational	14 V-A CPR excluded	ECMO restored systemic perfusion in bacterial sepsis; 86% of patients were successfully weaned; benefit in severe myocardial depression	70%
Bréchot N et al., Lancet 2020 [83]	Multicenter retrospective cohort study	82 V-A CPR excluded	ECMO as a rescue for sepsis-induced cardiogenic shock	51%
Banjas et al., J Intensive Care Med 2018 [84]	Retrospective observational	131 V-A, V-V, VAV CPR included	Early initiation, patient selection, and center experience are key determinants of outcome	42%
Falk et al., Crit Care Med 2019 [85]	Retrospective observational study	37 V-A (27), V-V (10) CPR excluded	Rising ECMO use in sepsis; high mortality in vasoplegic shock. Importance of phenotype guided selection	59.9% (90% for septic shock with LV failure, 64.7% in distributive shock)
Kim AK et al., ASAIO J 2023 [86]	Single-center retrospective analysis	246 mixed cardiogenic–septic shock 100 septic shock 466 cardiogenic shock V-A n.s.	Patients with mixed cardiogenic–septic shock had intermediate mortality between cardiogenic and septic shock	56.7% mixed cardiogenic–septic shock 49.6% cardiogenic shock 31% septic shock
Huang et al., J Thorac Cardiovasc Surg 2012 [87]	Single-center observational analysis	52 V-A post CPR included	Non-survivors were significantly older than survivors. Age ≥ 60 might be a contraindication	15%
Vogel DJ et al., Perfusion 2018 [88]	Single-center experience; retrospective analysis (VAV ECMO)	12 VAV n.s.	Hybrid VAV configuration improved oxygenation in septic cardiomyopathy	75%

Note: several studies and meta-analyses listed in this table partially include overlapping patient cohort. To avoid double counting, no pooled totals or aggregated survival rates were calculated.

**Table 4:** Outcomes of V-A ECMO in refractory septic shock: pediatric population. V-A ECMO: veno-arterial extracorporeal membrane oxygenation; CPR: cardiopulmonary resuscitation; n.s.: not specified whether post CPR patients were included.

Study/Year	Design	n	Main Findings	Reported Survival
Yang Y et al., Front Pediatr 2022 [9]	Systematic review and meta-analysis	535 V-A n.s.	Pooled pediatric survival 53% vs. 18% in adults; benefit greatest in early ECMO initiation and cardiac phenotype	53%
Melnikov et al., ASAIO J 2022 [10]	Retrospective single-center cohort study	31 V-A (21), V-V (10) CPR included	Higher survival in V-V (80%) vs. V-A (62%). Better outcomes in high-volume ECMO centers	71%
Ramanathan K et al., Crit Care 2020 [89]	Systematic review and meta-analysis (13 studies)	2054 Mainly V-A CPR included	Reversibility of septic cardiomyopathy	59%
MacLaren G et al., Pediatr Crit Care Med 2011 [90]	Retrospective case series (central cannulation)	23 Central V-A CPR included	Central cannulation for refractory shock; good post-ECMO cardiac recovery.	78%
MacLaren G et al., Pediatr Crit Care Med 2011 [91]	Single-center cohort	441 V-A (45) CPR included	ECMO improved survival in refractory pediatric sepsis; satisfactory long-term recovery	47%

Note: some pediatric series and meta-analyses may include overlapping cohorts from the same institutional registries. To avoid duplication, no pooled totals or combined survival percentages were calculated.

**Table 5:** Overview of adjunctive therapies and pharmacologic considerations under V-A ECMO supports. V-A ECMO: veno-arterial extracorporeal membrane oxygenation; Vd: volume of distribution; PK: pharmacokinetics; PD: pharmacodynamics; AKI: acute kidney injury; CRRT: continuous renal replacement therapy.

Modality	Primary Goals/Key Notes Under V-A ECMO	Evidence Summary/Practical Implication
Antimicrobial therapy	Early targeted killing; altered PK/PD on ECMO (circuit sequestration, expanded Vd)	Strong recommendation (moderate-quality evidence); cornerstone of sepsis management; early administration (<1 h) improves survival
Source control	Definitive eradication of infection focus; coordinate with anticoagulation	Strong recommendation (low-quality evidence); early, definitive intervention independently improves outcome
CRRT	Fluid, solute and acid–base control; AKI management during ECMO	Weak recommendation (moderate-quality evidence); effective for metabolic and volume control; no proven survival benefit
Hemoadsorption/hemoperfusion	Cytokine removal; vasoplegia modulation	No recommendation (very low-quality evidence); investigational, phenotype-dependent

**Table 6:** Key complications associated with V-A ECMO.

Complication	Underlying Mechanism/Risk Factors	Preventive Strategies
Bleeding	Systemic anticoagulation, thrombocytopenia, sepsis-related coagulopathy	Optimize anticoagulation targets (ACT, anti Xa), correct coagulopathy, monitor hemoglobin and drainage output
Thrombosis	Circuit stasis, suboptimal anticoagulation, platelet activation	Regular circuit surveillance; maintain appropriate anticoagulation; replace components early if thrombosis suspected
Neurological injury	Hypoxic–ischemic damage, embolic stroke, intracranial hemorrhage	Daily neurological assessment, NIRS monitoring
Limb ischemia	Femoral cannulation, vasopressor use, hypotension	Use a distal perfusion cannula; monitor NIRS and peripheral pulses
Infection	Prolonged cannulation, indwelling lines, immunosuppression	Strict asepsis, early diagnosis, appropriate antimicrobial coverage and source control
Post-ECMO syndrome	ICU-acquired weakness, neurocognitive deficits; psychological sequelae	Structured rehabilitation and long-term follow-up

**Table 7:** Key ethical and organizational considerations in V-A ECMO for refractory septic shock.

Domain	Key Issues	Practical Implications
Ethical proportionality	Invasive therapy with uncertain benefit; risk of non-beneficial prolongation of life	Weigh expected recovery against suffering and futility; apply multidisciplinary ethical review where feasible
Communication and consent	Prognostic uncertainty and emotional burden for families	Transparent discussion with surrogates about risks, benefits, and possible outcomes
Institutional preparedness	ECMO requires highly trained teams, 24/7 availability, and established protocols	Concentrate activity in high-volume centers to improve survival and safety
Resource stewardship	High costs and limited availability of circuits and trained staff	Implement equitable allocation policies and prioritize cases with reversible pathology and realistic recovery potential
Social equity	Geographic and economic disparities in ECMO access	Promote network collaboration and referral systems to ensure fairness in access to care

## Data Availability

No new data were created or analyzed in this study. Data sharing does not apply to this article.

## Abbreviations

The following abbreviations are used in this manuscript:

ECMO	Extracorporeal membrane oxygenation.
V-A ECMO	Veno-arterial extracorporeal membrane oxygenation.
LVEF	Left ventricular ejection fraction.
SCC	Surviving sepsis campaign.
CRRT	Continuous renal replacement therapy
